# Role of COX-2 in cough reflex sensitivity to inhaled capsaicin in patients with sinobronchial syndrome

**DOI:** 10.1186/1745-9974-6-7

**Published:** 2010-08-09

**Authors:** Yoshihisa Ishiura, Masaki Fujimura, Hiroki Yamamoto, Noriyuki Ohkura, Shigeharu Myou

**Affiliations:** 1 The Department of Internal Medicine, Toyama City Hospital, Toyama, Japan; 2 Respiratory Medicine, Cellular Transplantation Biology, Kanazawa University Graduate School of Medicine, Kanazawa, Japan

## Abstract

**Background:**

Sinobronchial syndrome is a cause of chronic productive cough. Inflammatory mediators are involved in the pathophysiology of chronic productive cough. Accumulating evidences indicate that cyclooxygenase (COX)-2, one of the inducible isoforms of COX, is a key element in the pathophysiological process of a number of inflammatory disorders. However, little is known about the role of COX-2 in chronic productive cough in patients with sinobronchial syndrome known as neutrophilic bronchial inflammation.

**Methods:**

The effect of etodolac, a potent COX-2 inhibitor, on cough response to inhaled capsaicin was examined in 15 patients with sinobronchial syndrome in a randomized, placebo-controlled cross-over study. Capsaicin cough threshold, defined as the lowest concentration of capsaicin eliciting five or more coughs, was measured as an index of airway cough reflex sensitivity.

**Results:**

The cough threshold was significantly (p < 0.03) increased after two-week treatment with etodolac (200 mg twice a day orally) compared with placebo [37.5 (GSEM 1.3) vs. 27.2 (GSEM 1.3) μM].

**Conclusions:**

These findings indicate that COX-2 may be a possible modulator augmenting airway cough reflex sensitivity in patients with sinobronchial syndrome.

## Background

Chronic productive cough is one of the most common symptoms in patients with sinobronchial syndrome, a common chronic bronchial disorder, which is defined as a coexisting chronic sinusitis and nonspecific chronic neutrophilic inflammation of the lower airways presenting with expectoration (e.g. chronic bronchitis, diffuse bronchiectasis and diffuse panbronchiolitis [1]). Although clinical efficacy for low-dose and long-term erythromycin therapy (EM therapy) has been established in patients with sinobronchial syndrome [2,3], our previous study has shown that 3-6 months are required to improve the cough, sputum and other symptoms [3]. So, it is important to clarify the mechanisms of chronic productive cough to improve social activity in patients suffering sinobronchial syndrome. Previous studies [2-5] implied the involvement of inflammatory mediators in sinobronchial syndrome, however, exact mechanisms underlying cough in this disorder has been remained obscure [3].

Cyclooxygenase (COX) is an essential enzyme in the pathway of prostaglandin formation from arachidonic acid. The previous studies [6,7] have revealed the existence of two isoforms of COX, namely COX-1 and COX-2, with similar molecular weights. COX-1 is a constituent of healthy cells and is expressed under normal conditions. On the other hand, COX-2 is highly inducible by a number of stimuli including cytokines and is associated with inflammation. It has been suggested that the induction and regulation of COX-2 may be key elements in the pathophysiological process of a number of inflammation [8]. These findings imply the role of COX-2 in controlling cough reflex sensitivity in sinobronchial syndrome, because cough is one of the major symptoms in this disorder. Our previous study showed that non-specific COX inhibitor, indomethacin, could modulate airway cough reflex sensitivity to inhaled capsaicin [9]. Therefore, we conducted this study in patients with sinobronchial syndrome, using etodolac, proven as a potent COX-2 inhibitor [10,11].

## Methods

### Subjects

Fifteen patients with stable sinobronchial syndrome (5 males and 10 females) with a mean age of 71.6 ± 1.3 (± SEM) (range 55-79) yrs participated in this study. All patients were lifetime nonsmokers or ex-smokers without exceeding 10 pack-years to exclude patient with COPD or smoking-induced bronchitis and with no history of viral infection for at least 4 weeks prior to the study. Informed consent was obtained from all subjects. This study was approved by the Ethics Committee of our hospital.

Sinobronchial syndrome is a common chronic bronchial disorder in Japan, which is not related to smoking. We provide some details, as it is not recognized as a diagnostic category by the ATS. Sinobronchial syndrome is defined as a coexisting chronic sinusitis and nonspecific chronic neutrophilic inflammation of the lower airways presenting with expectoration (e.g. chronic bronchitis, diffuse bronchiectasis and diffuse panbronchiolitis [1]). Suzaki et al. [12] reported that the sinobronchial syndrome was found in 10% of 309 patients with chronic sinusitis and in 55% of 74 patients with chronic lower respiratory tract infectious diseases. They suggested that there is a gene controlling the susceptibility to sinobronchial syndrome, especially diffuse panbronchiolitis, which is significantly associated with human leukocyte antigen (HLA)-BW54; this is found specifically in Japanese and not in Caucasians. The obstructive form of sinobronchial syndrome is known as "diffuse panbronchiolitis" [1].

Recognition of the sinobronchial syndrome is very important in Japan because long-term, low dose erythromycin therapy is specifically effective [2,3], as inhaled steroid therapy for bronchial asthma. In our patients, diagnosis of the sinobronchial syndrome was based on the following criteria: 1) productive cough on most days for at least 3 months for 2 consecutive years, 2) chronic sinusitis diagnosed based on symptoms (postnasal drip, nasal discharge and nasal obstruction), physical examinations and plain roentgenogram as indicated by opacities or air-fluid levels of one or more paranasal sinuses, 3) no history suggesting to the attending physician that they had bronchial asthma, 4) no history of wheezing syndrome, and 5) no significant emphysema documented by chest computed tomographic scan.

Each studied patient did not have perennial or vasomotor rhinitis. They were taking low-dose erythromycin and mucolytic agents, such as carbocysteine and ambroxol, however, not theophylline, β2-adrenoceptor stimulants, or glucocorticosteroids. This study was carried out when their symptoms were mild and stable.

### Assessment of cough reflex sensitivity to inhaled capsaicin

Cough receptor sensitivity was assessed by capsaicin provocation test [13]. Capsaicin (30.5 mg) was dissolved in Tween 80 (1 mL) and ethanol (1 mL) and then dissolved in physiological saline (8 mL) to make a stock solution of 1 × 10-2 M, which was stored at -20°C. This solution was diluted with physiological saline to make solutions starting at a concentration of 0.49 μM and increasing it by doubling concentrations up to 1000 μM. Each subject inhaled a control solution of physiological saline followed by progressively increasing concentrations of the capsaicin solution. Solutions were inhaled for 15 s every 60 s, by tidal mouth-breathing wearing a noseclip from a Bennett Twin nebulizer (3012-60cc, Puritan-Bennett Co., Carlsbad, California, USA). Increasing concentrations were inhaled until five or more coughs were elicited. The nebulizer output was 0.21 mL/min. The number of capsaicin-induced coughs was counted by a blinded medical technician in our pulmonary function laboratory. The cough threshold was defined as the lowest concentration of capsaicin that elicited five or more coughs.

### Study protocol

The concomitant medication was stopped at 9.00 p.m. on the previous day to allow a washout time of 12 h or more before the measurement of cough threshold to inhaled capsaicin at 10.00 a.m. on each test day.

Each patient attended 4 times separated by 2 weeks, at the same time each day. Control measurement of capsaicin cough threshold was carried out before the first treatment. After two weeks as wash out period, treatment with etodolac and placebo was performed in a randomized, cross-over fashion, putting a washout period of 2 weeks between the treatments. Etodolac tablet (200 mg) or its placebo was taken orally twice a day for 14 days and at 8.00 a.m. on the test day. FEV1 was measured on a dry wedge spirometer (Chestac 11, Chest Co., Ltd., Tokyo, Japan) before capsaicin challenge to assess the bronchoactive effect of the treatment regimens.

### Data analysis

Capsaicin cough threshold values were expressed as geometric mean with geometric standard error of the mean (GSEM). Forced vital capacity (FVC) and FEV1 were shown as arithmetic mean values ± SEM. The cough threshold, the FVC and the FEV1 values were compared between each pair of the four test periods (run-in, placebo treatment, wash out and etodolac treatment) by the Wilcoxon signed-ranks test. Data are transformed to logarithmic values for cough threshold at this test. A p-value of less than 0.05 was taken as significant.

## Results

**Figure 1:**
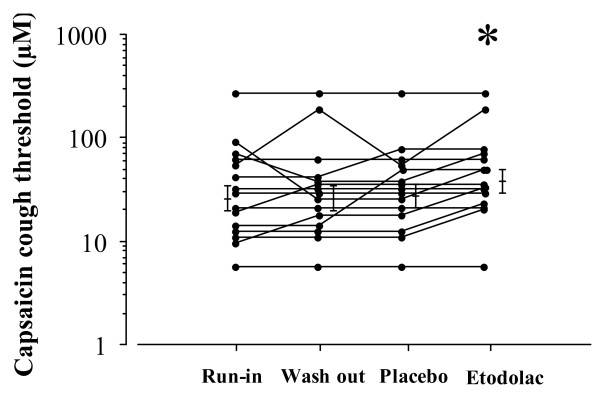
**Individual data of capsaicin cough threshold before each treatment and after placebo and etodolac treatments in patients with chronic bronchitis**. Each horizontal bar represents geometric mean value. * P < 0.03: an one-way analysis of variance using logarithmically transformed values.

Cough threshold to inhaled capsaicin before each treatment (run-in and washout period) and after treatment with etodolac and placebo are shown in figure 1. Geometric mean values for the cough threshold were 25.9 (GSEM 1.4) μM in run-in period, 25.9 (GSEM 1.4) μM in washout period, 27.2 (GSEM 1.3) μM after placebo treatment and 37.5 (GSEM 1.3) μM after etodolac treatment. The cough threshold after the etodolac treatment was significantly greater than the value after run-in period, wash out period and the placebo treatment (p < 0.03). FVC or FEV1 value was not significantly different among run-in period, washout period, etodolac treatment and placebo treatment as shown in the table 1.

**Table 1:** Pulmonary functions on etodolac and placebo treatments in patients with sinobronchial syndrome.

	Run-in	Placebo	Wash out	Etodolac
**FVCs as % pred. (%)**	106.7 ± 4.3	108.8 ± 4.1	106.6 ± 4.4	112.5 ± 1.2
**FEV1 s as % pred. (%)**	119.3 ± 5.1	118.8 ± 6.1	116.5 ± 9.1	112.0 ± 9.0
**FEV1/FVC ratio as % pred. (%)**	76.6 ± 6.0	74.6 ± 7.0	76.3 ± 6.4	72.5 ± 5.4

Data are shown as mean ± standard error of the mean for FVC, FEV1 and FEV1/FVC ratio. *p < 0.05 compared with each control value (Wilcoxon signed-ranks test).

Sputum cells were counted in seven patients and observed increasement of neutrophils (40-94%, mean 67.7%). CT scan was not conducted in this study, abnormal finding in sinus Xp were observed in every patients. After the administration of etodolac, none of the patients enrolled in this study complained of cardiovascular or gastroenterological symptoms which have been reported for other COX-2 inhibitors, such as rofecoxib, celecoxib and valdecoxib [14,15].

## Discussion

The present study showed that two-week treatment with a potent COX-2 inhibitor, etodolac, increased the cough threshold to inhaled capsaicin in stable patients with sinobronchial syndrome. No difference could be found in the baseline pulmonary function between etodolac and placebo treatments. From these findings, COX-2 may be a possible modulator augmenting airway cough reflex sensitivity in bronchitic airway.

Though cough is an important protective mechanism for the cleaning of the excessive mucus production [16], chronic cough can be a difficult clinical problem for physicians interfering with patient's quality of life through loss of sleep, interruption of work and social embarrassment. However, mechanism correlating to the cough reflex sensitivity in sinobronchial syndrome remains unclear.

Previous investigators demonstrated the efficacy of EM therapy for chronic bronchitic disorders; sinobronchial syndrome and diffuse panbronchiolitis, which is recognized as a severe obstructive form of sinobronchial syndrome [2,3]. EM therapy has excellent effect through the improvement of pulmonary inflammation by reducing the intrapulmonary chemotactic gradient or the ability of the neutrophils to respond to chemotactic factors, ultimately reducing the migration of neutrophils to inflammatory sites [2,3,17], but at least eight weeks are required to improve the symptoms including chronic productive cough [2,3]. We also failed to improve cough reflex sensitivity to inhaled capsaicin by four-week treatment of clarithromycin, another form of long term therapy for this disorder [18]. Thus it is important to clarify the potential mechanisms of chronic productive cough in patients suffering from sinobronchial syndrome to improve their symptoms more early.

COX is the key enzyme in the pathway of prostaglandin formation consisting of at least two isoforms, namely COX-1 and COX-2 [6,7]. COX-1 is constitutively expressed in most tissues, and maintains homeostasis of various physiologic functions. COX-2 is, with some exceptions, not generally found in healthy tissues, but its expression is markedly induced in inflammation. It can be induced by various stimuli, including inflammatory cytokines, resulting in further production of inflammatory substances such as prostanoids [6,7]. Previous study suggested that the induction and regulation of COX-2 may be key elements in the pathophysiological process of a number of inflammations [8]. We showed the modulating role of thromboxane, the family of metabolites resulting from enzymes possessing COX activity [19]. We also showed that non selective COX inhibitor, indomethacin, can modulate airway cough reflex sensitivity to inhaled capsaicin [9]. Recently, we conducted another study in patients with bronchial asthma [20], and showed the role of COX-2 for handling cough reflex sensitivity in asthmatic airway with chronic eosinophilic bronchial inflammation. We, therefore, conducted this study using etodolac with potent affinity for the COX-2 enzyme over the COX-1 enzyme, compared with that of celecoxib [10,11]. Unfortunately, we did not evaluate cough symptom scores and C2, but we clearly showed the beneficial effect of two-week treatment with etodolac for cough reflex sensitivity to inhaled capsaicin. So we can consider that COX-2 plays some roles in controlling cough reflex sensitivity in bronchitic airway with chronic neutrophilic bronchial inflammation, not only in asthmatic airway with chronic eosinophilic bronchial inflammation [20]. The precise mechanisms for modulating role of COX-2 in the pathophysiology of cough reflex remains unknown since we did not measure arachidonic metabolites in this study. Possible mechanism is that decreased sputum production caused by COX-2 inhibition may affect our result as shown in previous study [5]. Recently, Kamei and their colleagues [21] reported the effect of COX-2 inhibition in cough reflex sensitivity in guinea pigs and suggested that the inhibition of substance P release might result in the regulation of endogenous prostaglandins by COX-2 inhibitor on the capsaicin-sensitive sensory C-fibers. Therefore we can consider that COX-2, generated in chronic bronchitic airway known as neutrophilic inflammation [2-5,17], modulates airway cough reflex sensitivity through similar mechanisms. Another crucial problem in clinical practice remains about the cardiovascular risks of rofecoxib, celecoxib and valdecoxib in the placebo-controlled trials [22,23], however succeeding study did not found an elevated cardiovascular risk with etodolac [24]. Therefore we hope that adverse reactions in long-term should be clarified in future studies.

## Conclusions

In conclusion, the present study clearly showed that two week treatment with a potent COX-2 inhibitor, etodolac, attenuated cough reflex sensitivity to inhaled capsaicin in patients with sinobronchial syndrome. This is the first report indicating the modulating role of COX-2 in airway cough reflex sensitivity of bronchitic airway known as chronic neutrophilic inflammation. Further studies are required for elucidating the inflammatory process in bronchitic airways succeeding COX-2 induction.

## Abbreviations

ATS: American Thoracic Society; COX: cyclooxygenase; EM therapy: low-dose and long-term erythromycin therapy; FEV1: forced expiratory volume in one second; FVC: forced vital capacity; GSEM: geometric standard error of the mean; HLA: human leukocyte antigen; NSAIDS: nonsteroidal anti-inflammatory drugs.

## Competing interests

The authors declare that they have no competing interests.

## Authors' contributions

YI recruited the subjects, performed the data collecting and draft the manuscript. MF conceived the study, contributed to its design, data acquisition, data interpretation, and review and correction of the manuscript. HY performed the statistical analysis and data interpretation. NO participated in data acquisition. SM contributed to data interpretation. All authors have given final approval of the version to be published.
